# miR-589 promotes gastric cancer aggressiveness by a LIFR-PI3K/AKT-c-Jun regulatory feedback loop

**DOI:** 10.1186/s13046-018-0821-4

**Published:** 2018-07-16

**Authors:** Feifei Zhang, Kaitao Li, Mingxin Pan, Weidong Li, Juan Wu, Mingyi Li, Liang Zhao, Hui Wang

**Affiliations:** 1 Department of Pathology, Nanfang Hospital, Southern Medical University, Guangzhou, China; 2 Department of Pathology, School of Basic Medical Sciences, Southern Medical University, Guangzhou, China; 3 Second Department of Hepatobiliary Surgery, Zhujiang Hospital, Southern Medical University, Guangzhou, China; 4 Department of Medical Oncology, Affiliated Cancer Hospital & Institutes of Guangzhou Medical University, Guangzhou, China; 5 Radiotherapy Department, Affiliated Cancer Hospital & Institues of Guangzhou Medical University, Guangzhou, China

**Keywords:** miR-589, Gastric cancer, LIFR, Metastasis, Feedback loop

## Abstract

**Background:**

As novel biomarkers for various cancers, microRNAs negatively regulate genes expression via promoting mRNA degradation and suppressing mRNA translation. miR-589 has been reported to be deregulated in several human cancer types. However, its biological role has not been functionally characterized in gastric cancer. Here, we aim to investigate the biological effect of miR-589 on gastric cancer and to reveal the possible mechanism.

**Methods:**

Real-time PCR was performed to evaluate the expression of miR-589 in 34 paired normal and stomach tumor specimens, as well as gastric cell lines. Functional assays, such as wound healing, transwell assays and in vivo assays, were used to detect the biological effect of miR-589 and LIFR. We determined the role of miR-589 in gastric cancer tumorigenesis in vivo using xenograft nude models. Dual-luciferase report assays and Chromatin immunoprecipitation (ChIP) assay were performed for target evaluation, and the relationships were confirmed by western blot assay.

**Result:**

MiR-589 expression was significantly higher in tumor tissues and gastric cancer cells than those in matched normal tissues and gastric epithelial cells, respectively. Clinically, overexpression of miR-589 is associated with tumor metastasis, invasion and poor prognosis of GC patients. Gain- and loss-of function experiments showed that miR-589 promoted cell migration, metastasis and invasion in vitro and lung metastasis in vivo. Mechanistically, we found that miR-589 directly targeted LIFR to activate PI3K/AKT/c-Jun signaling. Meanwhile, c-Jun bound to the promoter region of miR-589 and activated its transcription. Thus miR-589 regulated its expression in a feedback loop that promoted cell migration, metastasis and invasion.

**Conclusion:**

Our study identified miR-589, as an oncogene, markedly induced cell metastasis and invasion via an atypical miR-589-LIFR-PI3K/AKT-c-Jun feedback loop, which suggested miR-589 as a potential biomarker and/or therapeutic target for the gastric cancer management.

**Electronic supplementary material:**

The online version of this article (10.1186/s13046-018-0821-4) contains supplementary material, which is available to authorized users.

## Background

Gastric cancer (GC) ranks the fourth in incidence among all cancers and second in cancer-associated mortality worldwide [1]. In 2015, approximately 952,000 patients were diagnosed with GC, and an estimated 723,000 patients died from GC, who were mainly from Asia [2]. Despite the improvement of diagnosis and treatment in recent years, new cases and estimated deaths continues to grow every year. 90% of GC patients are surgically curable at an early stage [3], but most patients are diagnosed in advanced stages with extensive invasion and lymphatic metastasis, which lead to poor prognosis with limited efficient treatment options [4, 5]. Therefore, elucidation of the mechanisms underlying GC metastasis and invasion will help to understand GC pathogenesis. In recent year, a great number of miRNAs have been identified and characterized as tumor suppressors or oncogenes in GC, many of which were associated GC metastasis and invasion. These findings suggest that miRNA might serve as potential biomarker for gastric carcinogenesis [6–8].

Increasing studies proved that microRNAs (miRNAs or miRs) played an important role in in human carcinogenesis [9]. miRNAs are a class of short noncoding RNAs (19~ 22 nucleotides) that regulate gene expression by arresting transcription or inducing transcriptional degradation, thereby mediating cellular function, such as cell proliferation, metastasis and invasion [10, 11]. According to a great number of miRNAs discovered, multiple miRNAs can regulate a particular mRNA while a single miRNA can target many mRNAs [12]. Moreover, miRNAs can be regulated by transcription factor which binds to its promoter region [13]. Intriguingly, the role of the same miRNA may be opposite in different cancer [14]. Therefore, more studies are needed to fully elucidate the function of the same miRNA in different cancer. Although miR-589 has been reported in lung cancer and hepatocellular carcinoma [15, 16], the biological effect of miR-589 in GC still remains unknown. Our study aims to determine the biological characteristic of miR-589 in GC and uncover the molecular mechanism underlying its function.

Leukemia inhibitory factor receptor (LIFR), a specific receptor for leukemia inhibitory factor (LIF), was firstly identified via expression screening of a placental cDNA [17]. Increasing evidence showed that LIFR played an important role in suppression of tumorigenesis. LIFR has been identified as a metastasis suppressor of breast cancer via the HIPPO-YAP pathway. Moreover, LIFR expression is down regulated in breast cancer and also significantly correlates with poor prognosis and overall survival outcomes of breast cancer patients [18–20]. Also, through the increased hypermethylation of its promoter, LIFR is observed to be markedly reduced in HCC tumor tissues [21, 22]. In GC, LIFR is positively regulated by LncRNA-LOWEG, which is a tumor suppressor inhibiting cell invasion [23]. Taken together, these findings suggest LIFR as prognostic marker for tumor progression, combination of LIFR and relevant genes are more efficient to evaluate prognoses.

In our studies, we investigated the biological function of miR-589 in GC, and addressed the underlying mechanism of miR-589-mediated cell migration, metastasis and invasion. Our data showed that miR-589 directly targeted LIFR, which blocked PI3K/AKT pathway and thus reduced c-Jun expression. Furthermore, c-Jun activated miR-589 transcription and thereby formed a 589-LIFR-PI3K/AKT-c-Jun loop. Our studies provide evidences supporting the existence of a novel feedback loop with a crucial role in tumor metastasis and invasion.

## Methods

### Cell culture and treatment

A series of GC cell lines (GSE-1, BGC823, MKN45, MGC803, AGS, MKN28) were obtained from Foleibao Biotechnology Development (Shanghai, China). The cells were cultured in RPMI 1640 medium (Gibco, Grand Island, NY, USA) supplemented with 10% fetal bovine serum (FBS) (NBCS) (PAA Laboratories, Inc., Pasching, Austria). All of these cell lines were incubated in a humidified chamber with 5% CO_2_ at 37 °C. For inhibitor treatment, 10 mmol/L PI3K inhibitor LY294002 (Cell Signal Technology, Danvers, MA) was added in the cultured cells every two days.

LIFR plasmids, miR-589 mimic, anti-miR-589 oligos and all siRNA oligos including c-Jun specific siRNAs (si-1973, si-1554, si-2358 and si-1113) were purchased from GenePharma (Shanghai, China). GC cells at exponential growth phase were plated into 6-well plates for 24 h at a density of 0.5 × 10^5^ cells/mL, and transfected with 1 mg of siRNA or 4 μg cDNA using Lipofectamine 2000 reagent (Invitrogen; Carlsbad, Calif, USA) in reduced serum medium (OPTI-MEM-I; Invitrogen) according to the manufacturer’s protocol.

### Clinical samples

Fresh primary GC specimens with paired normal gastric tissues were obtained from the Tumor Tissue Bank of Nanfang Hospital. In each case, pathological diagnosis was made after elective surgery for GC in Nanfang Hospital during 2009 and 2014. All experiments performed are endorsed by the Ethics Committee of Southern Medical University and complied with the Declaration of Helsinki. No informed consent was required because data were going to be analyzed anonymously.

### Animals

All animal experiments were carried out with the approval of the Southern Medical University Animal Care and Use Committee in accordance with the guidelines for the ethical treatment of animals. Nude nu/nu mice were maintained in a barrier facility in racks filtered with high-efficiency particulate air filter. The animals were fed with an autoclaved laboratory rodent diet. The mice in this study were purchased from the Experimental Animal Centre of Southern Medical University, which is certified by the Guangdong Provincial Bureau of Science. All animal experiments involved ethical and humane treatment under a license from the Guangdong Provincial Bureau of Science.

### Western blot analysis

Protein expression was assessed by immunoblot analysis of cell lysates (20–40 μg) in RIPA buffer in the presence of rabbit antibodies to GAPDH (1:1000; Santa Cruz, California, USA); mouse antibody to LIFR (1:1000; Proteintech); rabbit antibodies to PI3K, p-PI3K, p-AKT(Ser473)(#4060), AKT(#4691) (1:1000; CST, Danvers, MA). Relative protein abundance of phosphorate proteins was determined by normalisation with levels of corresponding total protein. Relative protein abundance of total protein was determined by normalisation with levels of corresponding endogenous control protein.

### Statistical analysis

Data were analyzed using SPSS version 19.0 software (SPSS; Chicago, USA). Statistical significance of difference between groups was determined by a two-tailed paired Student’s *t* test. Kaplan-Meier plots were performed to investigate the prognostic relevance of PIK3AP1 in univariate analysis. Statistical significance was established at *P* < 0.05.

## Results

### miR-589 is up-regulated in GC tissues and cell lines and associated with poor prognosis of patients with GC

**Fig. 1:**
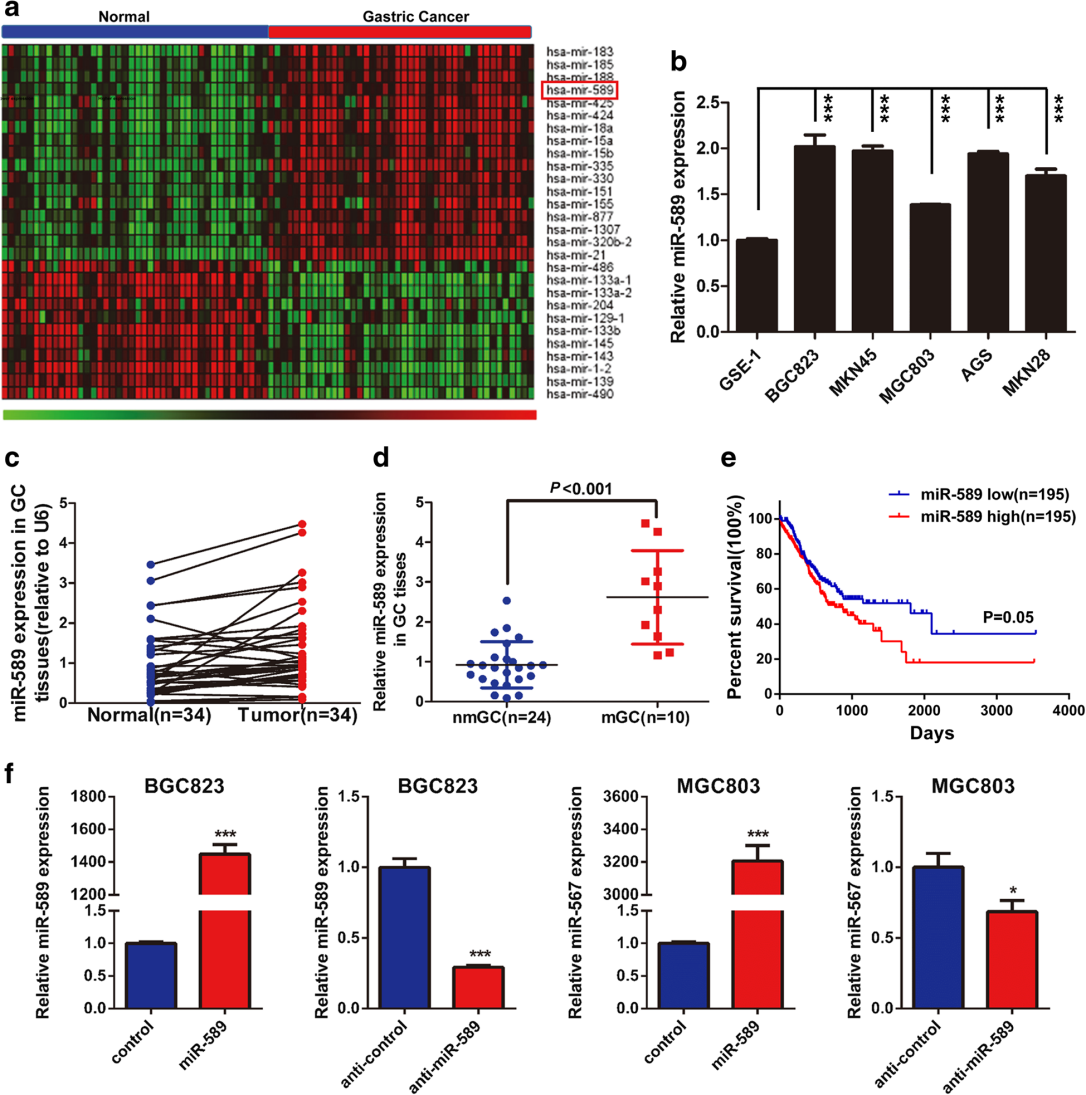
miR-589 is up-regulated in GC tissues and cell lines and associated with poor prognosis of patients with GC. **a** Data from TCGA showed miR-589 expression was markedly increased in GC tissues compared with normal tissues. **b** Relative expression levels of miR-589 in gastric cancer cell lines and a normal gastric epithelial cell line, GES-1, Student’s t-test, mean ± SD, ***P < 0.001. **c** The expression level of miR-589 was upregulated in GC tissues compared with corresponding non-cancer tissues. **d** The expression level of miR-589 was upregulated in metastatic GC (mGC) tissues compared with non-metastatic GC (nmGC) tissue. **e** Analysis of TCGA data showed Kaplan–Meier survival curves for GC patients with distinct expression level of miR-589. **f** Real-time PCR was performed to detect the mRNA expression of miR-589 in MGC803 and BGC823 cells, both transfected with miR-589 mimic or anti-miR-589, Student’s t-test, mean ± SD, *P < 0.05; ***P < 0.001

The microarray result showed that miR-589 was upregulated an average of 1.25 folds (*P* < 0.001) in GC tumor tissues compared with adjacent normal tissues (Fig. 1a). We subsequently detected miR-589 mRNA expression in GC cell lines and normal gastric epithelium cell line GES-1. Real-time PCR assay showed higher level of miR-589 expression in five GC cell lines compared with GES-1 cell line (Fig. 1b). Consistent with this result, we found miR-589 is markedly up-regulated in GC tissues compared with matched normal tissues (Fig. 1c). As compared to tumor tissues without metastasis, metastatic tumors tissues showed relatively high miR-589 expression (Fig. 1d, *P* < 0.001). These data suggested miR-589 as an oncogene in GC tumorigenesis and metastasis. To evaluate the clinical relevance of miR-589 expression, we analyzed its relationship with pathological features. As shown in Additional file 1: Table S1, miR-589 expression in GC tissues was positively correlated with depth of tumor invasion (*P* = 0.016) and distant metastasis (*P* = 0.001), but had no correlation with the age and gender. Subsequently, we used TCGA data for survival analysis, the Kaplan-Meier survival curves displayed a significant trend towards poorer survival for patients whose tumors showed high miR-589 expression, compared with those tumors showed low miR-589 expression (Fig. 1e; *P* = 0.05).

### Exogenous miR-589 induces GC cell migration and invasion in vitro

**Fig. 2:**
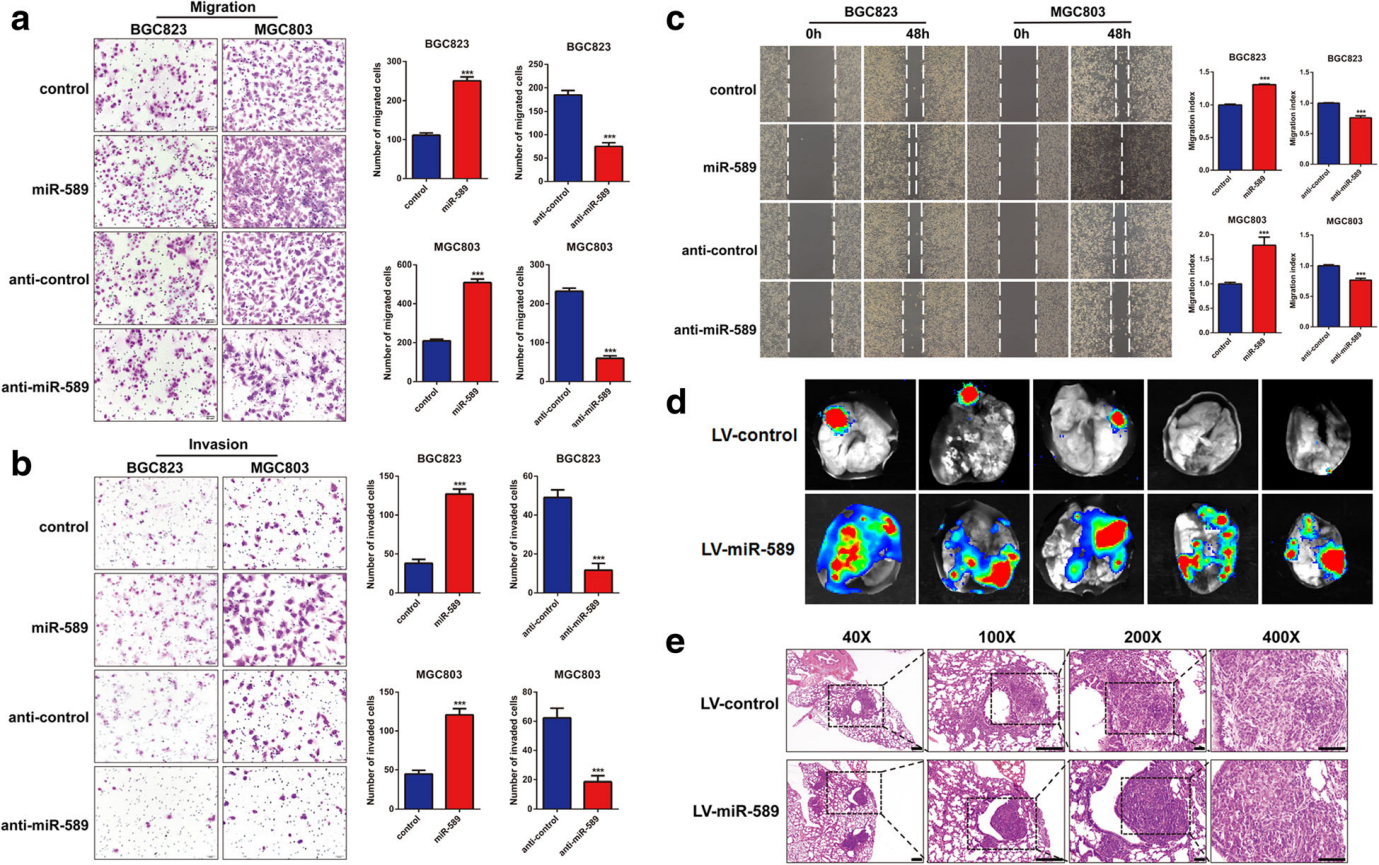
miR-589 induces GC cell migration, metastasis and invasion in vitro and promotes GC cell metastasis in vivo. Metastasis (**a**) and invasion (**b**) in MGC803 and BGC823 cells increased after transfected with miR-589 and decreased after transfected with anti-miR-589 in a transwell assay, Student’s t-test, mean ± SD, ***P < 0.001. **c** Wound healing was promoted in miR-589-transduced cells and delayed in anti-miR-589-transduced cells after 48 h. Results were expressed as mean ± SD of migrated distance from three different points. Migrated distance = width (0 h) – width (48 h), ***P < 0.001. **d** In vivo metastasis assays using bioluminescence imaging to detect metastases of MGC803-luciferase cells transfected with Lv-miR-589 or Lv-control. **e** Representative hematoxylin and eosin staining of lung tissue from different experimental groups. Scale bar = 50 μm

In order to investigate the biological effect of miR-589 on GC cells, we chose MGC803 and BGC 823 cell lines to perform the gain- or loss-of function. We transfected miR-589 mimic oligonucleotides and anti-miR-589 into MGC803 and BGC823 cell lines. Real-time PCR assay confirmed the transfection efficiency (Fig. 1f). Wound healing and trans-well assays showed that miR-589-knockdown cells exhibited reduced migratory, metastatic and invasive capability, whereas an opposite result was observed in miR-589-overexpressing cells (Fig. 2a-c). Together, miR-589 played a positive role in GC cell migration, metastasis and invasion.

### Endogenous overexpression of miR-589 promotes metastasis of GC cell in vivo

To evaluate the in vivo effect of miR-589 on GC cell metastasis, we established miR-589 stably expressing MGC803 cells by lentivirus injection. MGC803 cells stably overexpressing miR-589 were injected into nude mice via the lateral tail vein along with control cells. The bioluminescence images showed that miR-589 can significantly promote lung metastasis (Fig. 2d). The mice were sacrificed after 8 weeks and their lungs were dissected. Haematoxylin and eosin staining was conducted to evaluate the tissue morphology. We evaluated the number of lung metastasis nodules and found they were markedly increased in the MGC803-miR-589 group compared with the control group (Fig. 2e). These findings suggest that miR-589 increased GC metastasis and thus may act as a potential therapeutic target against metastatic GC.

### miR-589 involves in PI3K/AKT signal pathway in GC progression

**Fig. 3:**
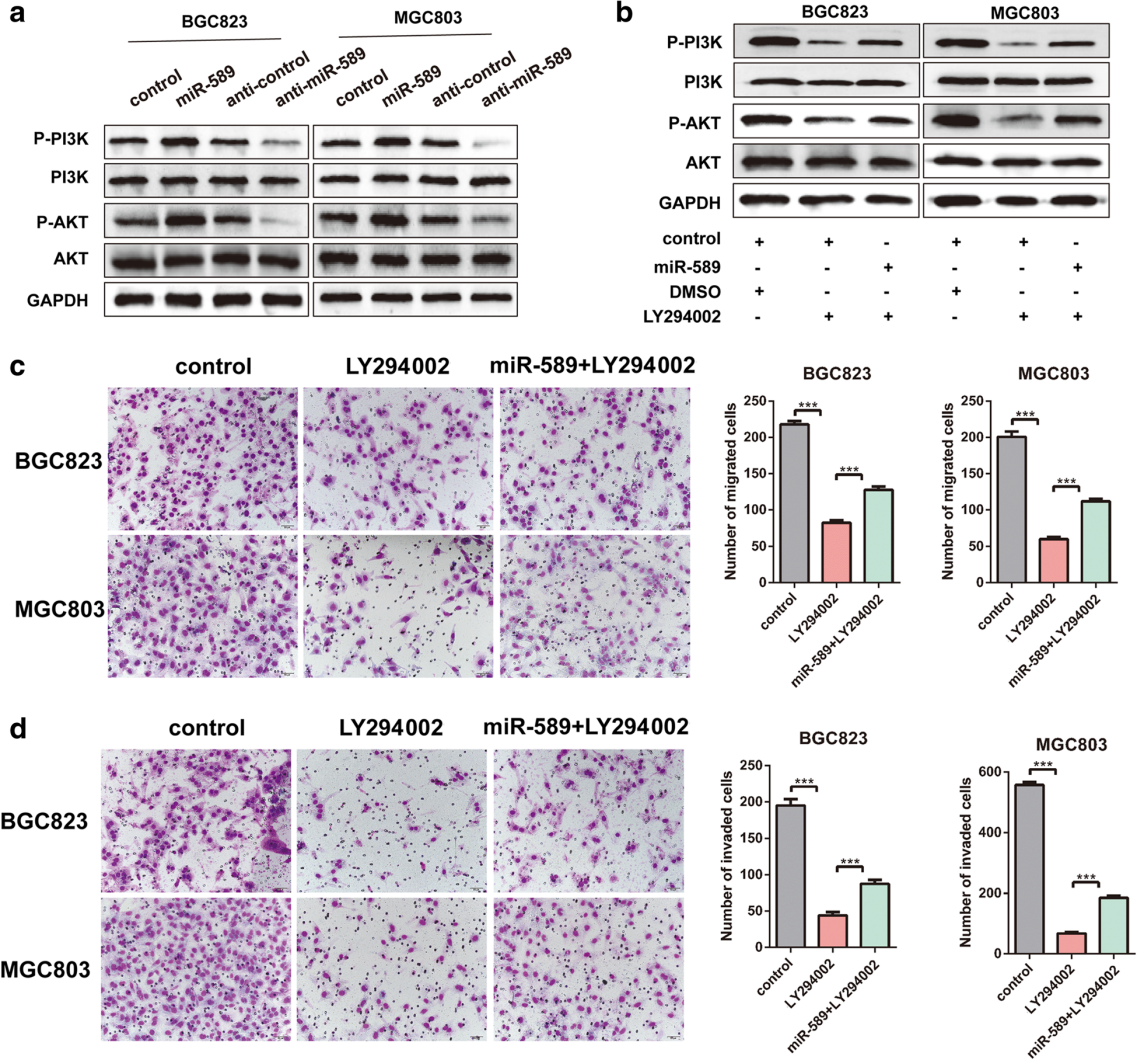
miR-589 involves in PI3K/AKT pathway in GC. **a** Western blot experiments were used to analyze the expression of relevant proteins in PI3K/AKT signal pathway and LIFR after miR-589 overexpression and knockdown in MGC803 and BGC823 cells. **b** The downregulation of p-AKT by LY294002 were abrogated after administration of miR-589 in MGC803 and BGC823 cells. The LY294002-supressed cell migration (**c**) and invasion (**d**) were counteracted by miR-589 in MGC803 and BGC823 cells, Student’s t-test, mean ± SD, ***P < 0.001

Western blot analysis showed that miR-589 overexpression was positively correlated with PI3K and AKT phosphorylation. In contrast, knockdown of miR-589 significantly blocked AKT/PI3K pathway (Fig. 3a). These results suggested miR-589 as an upstream regulator of PI3K/AKT pathway. LY294002 was an inhibitor which was used to block AKT signaling pathway, western blot showed miR-589 significantly compensated the effect of LY294002 (Fig. 3b), indicating that miR-589 positively regulated AKT pathway. Meanwhile, miR-589 was sufficient to compensate the effects of LY294002 on GC cell migratory and invasive abilities (Fig. 3c & d). Taken together, miR-589 plays an important role in the activation of PI3K/AKT pathway, which may explain part of the mechanism underlying miR-589 function.

### LIFR is a direct target of miR-589

**Fig. 4:**
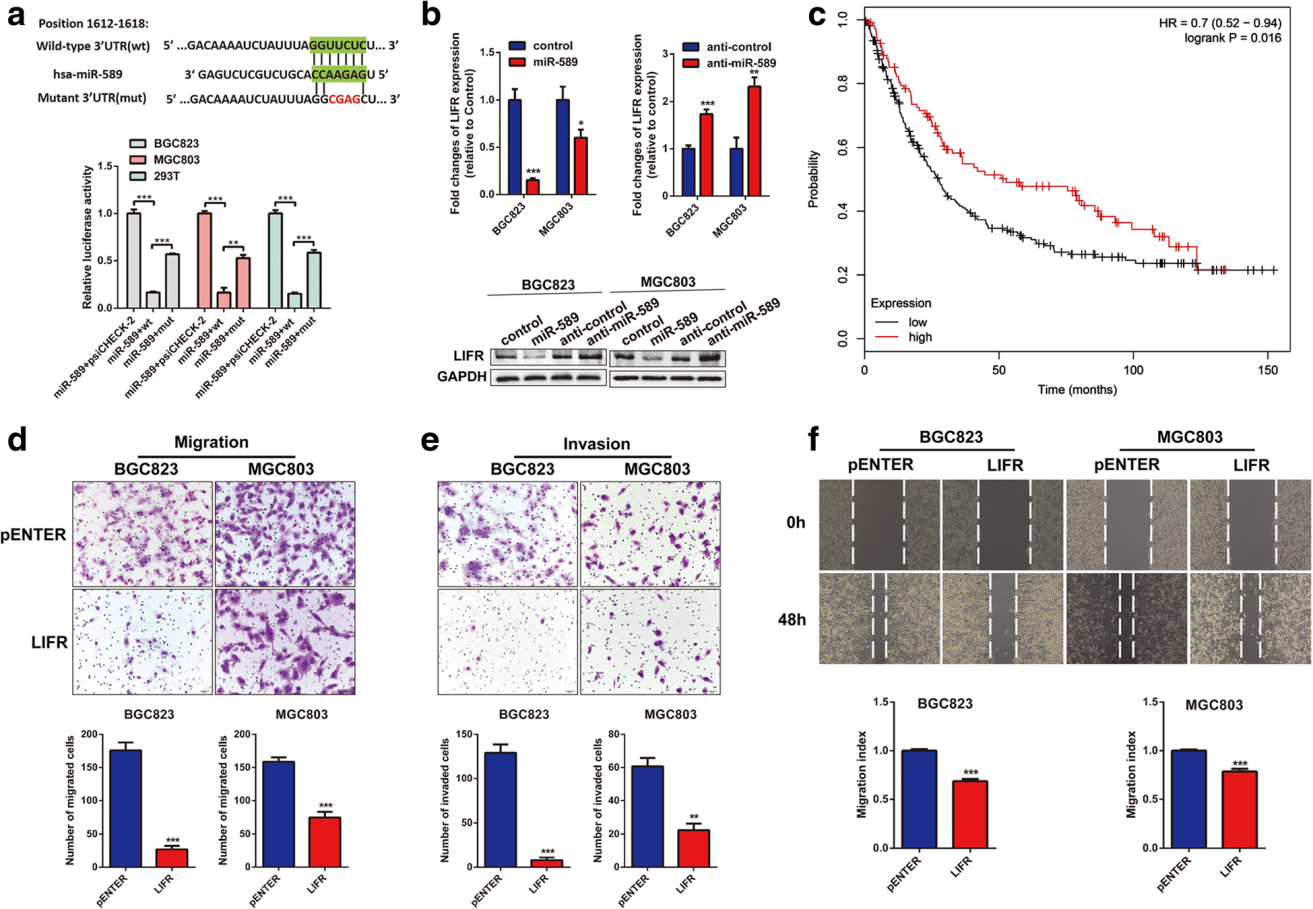
LIFR is the direct target of miR-589 and suppresses GC cell metastasis, invasion and migration. **a** miR-589 and its putative binding sequences in the 3’UTR of LIFR. A mutation was generated in the complementary site that bound to the seed region of miR-589. Luciferase reporter assay was used to determine miR-589 direct targeting the LIFR 3’UTR, Student’s t-test, mean ± SD, **P < 0.01; ***P < 0.001. **b** Real-time PCR and Western blot analysis were performed to detect the mRNA and protein expression of LIFR in MGC803 and BGC823 cells, both transfected with miR-589 mimic or anti-miR-589, Student’s t-test, mean ± SD, *P < 0.05; **P < 0.01; ***P < 0.001. **c** Kaplan-Meier survival plots showed that lower expression of LIFR resulted in a worse survival, HR = 0.7, *P* = 0.016. Transwell assay (**d** & **e**) and wound healing assay (**f**) of MGC803 and BGC823 cells were performed after transfected with LIFR or pENTER vector, Student’s t-test, mean ± SD, **P < 0.01; ***P < 0.001

Bioinformatics approach based on the database TargetScan predicted that LIFR was the putative target of miR-589. The analysis of the 3’-UTR of LIFR mRNA revealed the potential binding sites for miR-589, which implied the existence of a regulative relationship between miR-589 and LIFR. Dual-luciferase reporter assays were performed to prove miR-589 regulation of LIFR. miR-589 markedly decreased the luciferase activity of wide-type LIFR 3’-UTR in both MGC803 and BGC823 cells, whereas the suppression effect was abrogated after the 3’-UTR binding site of LIFR was mutated (Fig. 4a). Next, Real-time PCR analysis showed an increased LIFR expression level in miR-589-knockdown cells, and a decreased LIFR level in miR-589-overexpressing cells. Correspondingly, LIFR protein levels coincided with the change of mRNA levels in miR-589-knockdown and miR-589-overexpressing cells (Fig. 4b). Therefore, we drew a conclusion that LIFR is the direct target of miR-589.

### LIFR suppresses GC cell migration and invasion

Given that miR-589 targeted LIFR and suppressed its expression, we investigated the biological function of LIFR in GC cells. Data from Kaplan-Meier plotter database (http://kmplot.com/analysis/) were utilized to visualize the association between LIFR expression and overall survival in GC patients. The results showed patients with high LIFR expression in tumors had a trend towards better survival when compared with patients showed low LIFR expression (Fig. 4c; HR = 0.7, *P* = 0.016). To evaluate the effects of LIFR on cellular process, we performed wound healing and transwell assays in MGC803 and BGC823 cells. LIFR-overexpressing cells displayed opposite phenotype compared with miR-589-overexpressing cells and showed markedly inhibited cell migratory and invasive abilities (Fig. 4d-f).

### LIFR is essential to miR-589-mediated promotion of GC cell behavior and PI3K/AKT signaling activation

**Fig. 5:**
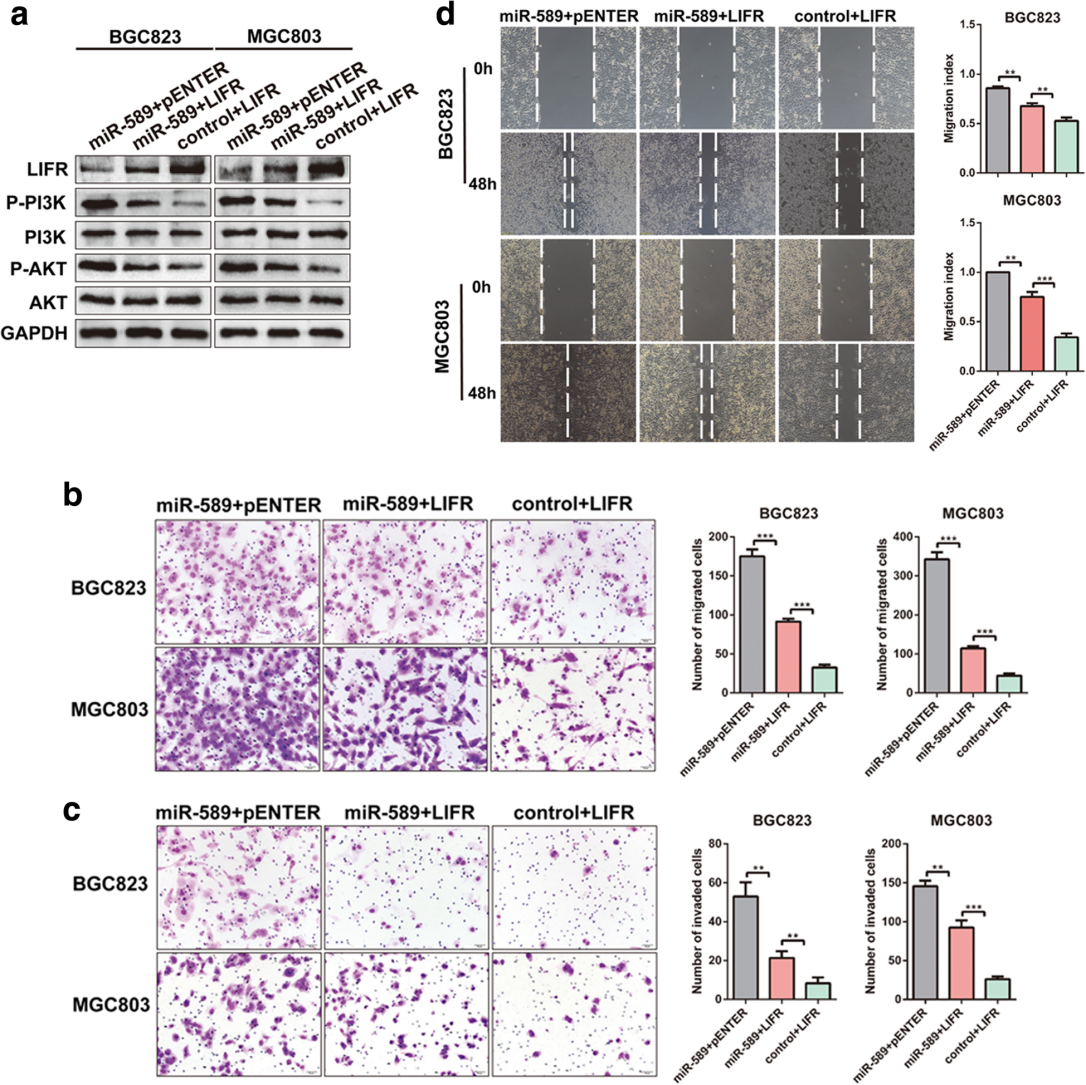
LIFR is essential to miR-589-mediated promotion of GC cell behavior and PI3K/AKT signaling activation. **a** The upregulation of relevant proteins in PI3K/AKT signal pathway and downregulation of LIFR induced by miR-589 were abrogated after administration of LIFR in MGC803 and BGC823 cells. Transwell assay (**b** & **c**) and wound healing assay (**d**) showed miR-589-induced cell metastasis, invasion and migration were counteracted after administration of LIFR, Student’s t-test, mean ± SD, **P < 0.01; ***P < 0.001

Western blot exhibited that LIFR efficiently revered miR-589-induced increase of p-PI3K and p-AKT (Fig. 5a), suggesting miR-589 regulates PI3K/AKT pathway via targeting LIFR. Subsequent rescue experiments showed that transiently transfecting LIFR into miR-589-overexpressing GC cells significantly weakened miR-589-midiated promotion of cell migration, metastasis and invasion (Fig. 5b-d). Collectively, these data indicate that LIFR attenuates miR-589-induced cell behavior and PI3K/AKT signaling activation.

### C-Jun stimulates transcriptional activity of miR-589 by binding to its promoter region

**Fig. 6:**
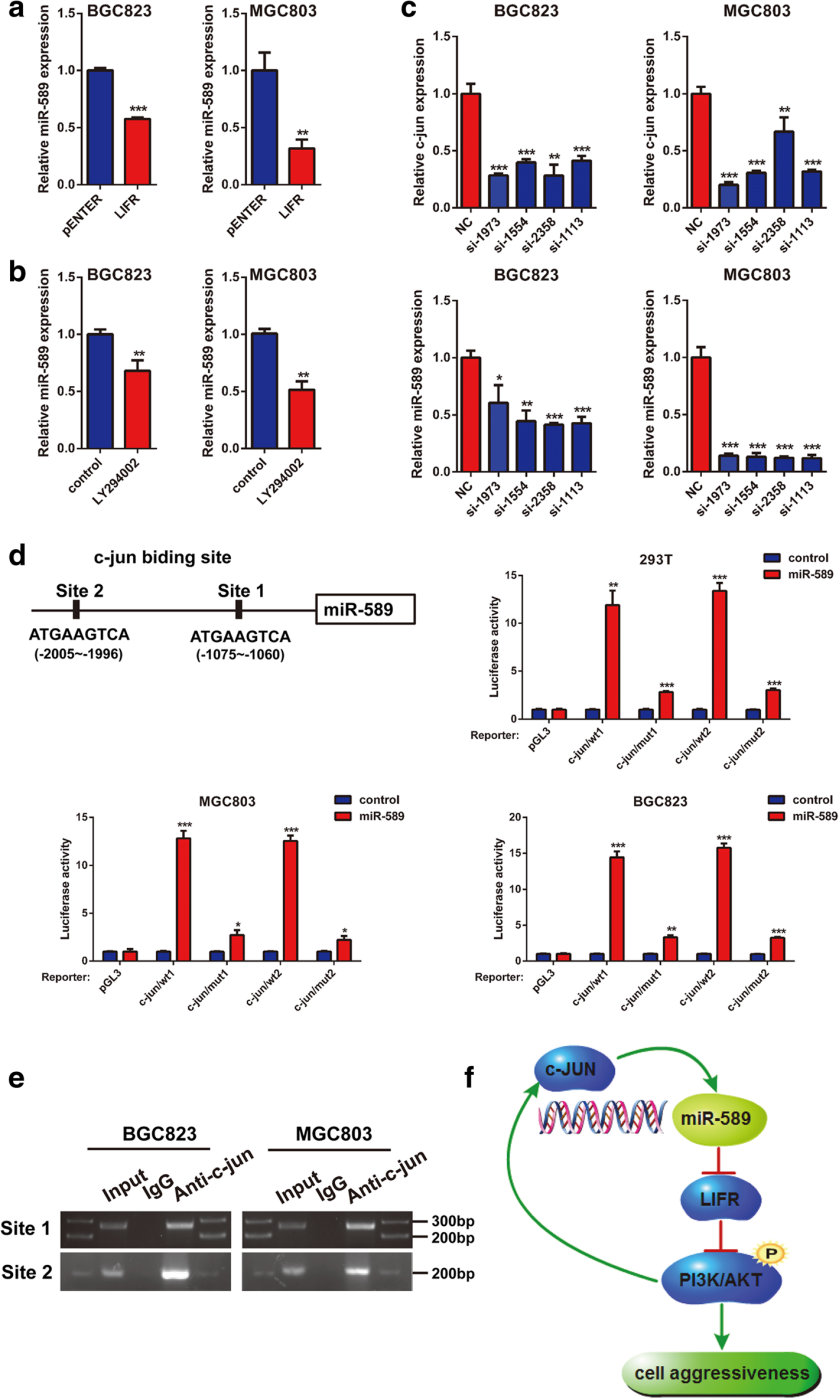
c-Jun stimulates miR-589 expression by binding to its promoter region. **a** & **b** Real-time PCR was performed to detect the mRNA expression of miR-589 in MGC803 and BGC823 cells treated with LIFR and LY294002, Student’s t-test, mean ± SD, **P < 0.01; ***P < 0.001. **c** Knocking down c-Jun expression by siRNAs inhibited miR-589 and c-Jun expression in MGC803 and BGC823 cells, Student’s t-test, mean ± SD, *P < 0.05; **P < 0.01; ***P < 0.001. **d** Schematic representation of the promoter regions of miR-589 with the putative c-Jun TFBSs. Relative luciferase activity of the indicated promoter vectors in 293 T, MGC803 and BGC823 cells transfected with c-Jun plasmids, Student’s t-test, mean ± SD, *P < 0.05; **P < 0.01; ***P < 0.001. **e** PCR gel showing amplification of c-Jun-binding sites after ChIP using antibody against c-Jun. The gel figures were accompanied by the locations of molecular weight markers. **f** A schematic for an atypical miR-589-LIFR–PI3K/AKT-c-Jun feedback loop

Transcriptional factor c-Jun is known to be an downstream target of AKT signaling [24], intriguingly, we used JASPAR bioinformatics database to analyze the promoter region of miR-589 and found two putative c-Jun-binding sites (from − 2005 to − 1996 and from − 1075 to − 1060) within the region (Fig. 6d). Therefore, we predicted that c-Jun inversely regulated miR-589 and formed a miR-589-LIFR-PI3K/AKT-c-Jun feedback loop. Subsequently, we used 4 specific c-Jun siRNAs to interfere c-Jun expression in MGC803 and BGC823 cell lines. Indeed, Real-time PCR analysis showed a relatively low miR-589 expression in cells treated with c-Jun siRNAs (Fig. 6c). Moreover, LY294002 and LIFR overexpression significantly decreased miR-589 expression (Fig. 6a & b), suggesting the existence of a miR-589-LIFR-PI3K/AKT-c-Jun regulatory feedback loop.

Chromatin immunoprecipitation (ChIP) assays was performed to confirm the relationship between c-Jun and miR-589. DNA from the immunoprecipitated chromatin displayed a significant enrichment of the predicted region compared with negative control (IgG) pulldown (Fig. 6e). Furthermore, an increase of the wild-type miR-589 promoter luciferase activity was observed on upregulation of c-Jun in 293 T, BGC823 and MGC803 cell lines (Fig. 6d; *P* < 0.001). Thus, c-Jun increased miR-589 expression via binding to its promoter region. Collectively, these results offer a conclusion that miR-589 induced cell migration, metastasis and invasion via a miR-589-LIFR-PI3K/p-AKT-c-Jun feedback loop.

## Discussion

Although metastasis and invasion are the overwhelming causes of cancer mortality, a comprehensive picture of modular and cellular determinants governing these processes remains largely unexplored [9]. Multiple lines of evidence has proved that abnormal expression of miRNAs was closely correlated with cancer migration, metastasis and invasion [9, 25]. In our studies, miR-589 expression was obviously upregulated in GC tissues compared with normal tissues. Subsequent experiment showed that miR-589 endowed GC cells with a more invasive phenotype as well as enhanced migratory and metastatic ability. Thus, miR-589 may serve as an attractive candidate biomarker or therapeutic target for GC progression.

Recent work has revealed LIFR function is regulated by miRNAs including miR-629-3p, miR-200b and miR-9 [26–28]. In agreement with these findings, our study showed an inverse expression of LIFR in relation to miR-589 in GC cells. To verified molecular interaction between LIFR and miR-589, we performed luciferase reporter assay which indicated that miR-589 directly targeted LIFR and reduced its expression. The association of the miR-589 expression with LIFR antitumor function highlighted the important role of miRNAs in tumorigenesis.

As a member of gp130 receptor family, LIFR is architecturally similar to gp130, which is an interleukin-6 signal transducer (IL6ST) [29]. After LIFR forms multimeric complexes with gp130, LIF binds the complexes and subsequently activated JAK/STAT, MAPK, and PI3K/AKT signaling pathway [30]. However, LIFR is found to block PI3K/AKT pathway in hepatocellular carcinoma (HCC) [31]. Consistent with this finding, we observed a weaker phosphorylation of PI3K and AKT in GC cells overexpressing LIFR. Therefore, LIFR may play a contradictory or conflicting role in PI3K/AKT pathway in different cells. Future studies concerning LIFR-mediated PI3K and AKT phosphorylation are needed to fully elucidate the mechanism underlying LIFR function.

The AP-1 family member c-Jun is a well-known oncogene which has been linked to metastatic and invasive properties of various cancers [32–34]. For example, high expression of c-Jun was observed in breast cancer tumors with invasive phenotype. Breast cancer cells with ectopic overexpression of c-Jun showed increased motility and invasiveness [35–37], these findings demonstrated a critical role for c-Jun in cancer cells migration and invasion characteristics. In our study, we proved that miR-589 expression was positively correlated with c-Jun expression, which suggested the contribution of c-Jun to miR-589-induced cells migration, metastasis and invasion.

c-Jun N-terminal kinases (JNK) were firstly identified as kinases responsible for binding and phosphorylating c-Jun at two regulatory sites (Ser-63 and Ser-73) located within its transcriptional activation domain [38]. As a member of a subgroup of MAPK signaling pathway, JNK signaling pathway is one of the crucial downstream signaling pathway of AKT [24]. In summary, AKT acted as an upstream regulator of JNK and c-Jun and induced c-Jun expression via an AKT/JNK/c-Jun signaling. In agreement with this findings, we found that treatment with LY294002 triggered a decrease in c-Jun expression in GC cells, whereas, we did not detect the change of JNK expression after using LY294002. Therefore, extensive study is required to examine whether LY294002 reduce c-Jun expression through AKT/JNK/c-Jun signaling.

As a transcription factor, c-Jun functions as an upstream regulator of many genes, including miRNAs, and participates in various signaling pathway [39]. Recent study has showed that c-Jun stimulated some key miRNAs transcription via binding to its promoter region. Inversely, c-Jun expression was modulated by the same miRNA, thus formed a feedback loop and regulated its own expression [40]. In our study, bioinformatics analysis predicted miR-589 as the target of c-Jun. Subsequent experiments proved that c-Jun activated miR-589 transcription via binding to the predicted sites within its promoter region. In turn, miR-589 affected c-Jun expression by a miR-589/LIFR/PI3K/AKT pathway. Collectively, these results offer an atypical miR-589-LIFR-PI3K/AKT-c-Jun feedback loop explaining the mechanism underlying miR-589 function.

## Conclusions

As summarized in our model in Fig. 6f, miR-589 is not only an upstream regulator but also a key target in the pathway we discovered. Experiments showed that miR-589 stimulate the phosphorylation of AKT by directly targeting LIFR and resulted in suppression of LIFR expression. Correspondingly, as a downstream of AKT pathway, c-Jun expression was promoted together with the activation of AKT pathway. Interestingly, c-Jun inversely bound to the promoter region of miR-589 and activated its transcription. Therefore, miR-589 does not induce gastric carcinogenesis alone, instead, miR-589 forms a miR-589-LIFR-PI3K/AKT-c-Jun feedback loop which contributes to cell migration, metastasis and invasion. Taken together, our findings provide a mechanistic explanation for miR-589 biological effects on GC, and suggest miR-589 as a biomarker and/or therapeutic target for GC progression.

## Additional file

Additional file 1: Supplementary materials and methods. (DOC 82 kb)

## Abbreviations

AKT: protein kinase B
ChIP: Chromatin immunoprecipitation
FBS: fetal bovine serum
GC: gastric cancer
IHC: immunohistochemistry
LIFR: leukemia inhibitory factor receptor
miR-589: microRNA-589
miRNA: microRNA
NC: negative control
PCR: polymerase chain reaction
PI3K: phosphatidylinositol-4,5-bisphosphate 3-kinase
siRNA: small-interfering RNAs
UTR: untranslated region

## References

[1] Van Cutsem E (2016). Gastric cancer. Lancet.

[2] Shah M (2015). Update on metastatic gastric and esophageal cancers. J Clin Oncol.

[3] Wang J (2012). Treatment strategy for early gastric cancer. Surg Oncol.

[4] Wagner A (2006). Chemotherapy in advanced gastric cancer: a systematic review and meta-analysis based on aggregate data. J Clin Oncol.

[5] Mirzaei H (2016). Circulating microRNAs as potential diagnostic biomarkers and therapeutic targets in gastric Cancer: current status and future perspectives. Curr Med Chem.

[6] Wang M (2014). Deregulated microRNAs in gastric cancer tissue-derived mesenchymal stem cells: novel biomarkers and a mechanism for gastric cancer. Br J Cancer.

[7] Cui L (2013). Gastric juice MicroRNAs as potential biomarkers for the screening of gastric cancer. Cancer.

[8] Carlomagno N (2017). Diagnostic, predictive, prognostic, and therapeutic molecular biomarkers in third millennium: a breakthrough in gastric Cancer. Biomed Res Int.

[9] Di Leva G, Garofalo M, Croce C (2014). MicroRNAs in cancer. Annu Rev Pathol.

[10] Bartel D (2004). MicroRNAs: genomics, biogenesis, mechanism, and function. Cell.

[11] Li J (2014). MicroRNAs as novel biological targets for detection and regulation. Chem Soc Rev.

[12] Song J, Meltzer S (2012). MicroRNAs in pathogenesis, diagnosis, and treatment of gastroesophageal cancers. Gastroenterology.

[13] Martinez N, Walhout A (2009). The interplay between transcription factors and microRNAs in genome-scale regulatory networks. Bioessays.

[14] Ma D (2017). miR-93-5p/IFNAR1 axis promotes gastric cancer metastasis through activating the STAT3 signaling pathway. Cancer Lett.

[15] Liu C (2017). Hypermethylation of miRNA-589 promoter leads to upregulation of HDAC5 which promotes malignancy in non-small cell lung cancer. Int J Oncol.

[16] Zhang X (2016). miR-589-5p inhibits MAP3K8 and suppresses CD90(+) cancer stem cells in hepatocellular carcinoma. J Exp Clin Cancer Res.

[17] Gearing D (1991). Leukemia inhibitory factor receptor is structurally related to the IL-6 signal transducer, gp130. EMBO J.

[18] Chen, D.H., et al., LIFR is a breast cancer metastasis suppressor upstream of the Hippo-YAP pathway and a prognostic marker. Vol. 18. 2012. 10.1038/nm.2940 PMC3684419 23001183

[19] Hergovich A (2012). YAP-Hippo signalling downstream of leukemia inhibitory factor receptor: implications for breast cancer. Breast Cancer Res.

[20] Zeng H (2016). Feedback activation of leukemia inhibitory factor receptor limits response to histone deacetylase inhibitors in breast Cancer. Cancer Cell.

[21] Blanchard F (2003). DNA methylation controls the responsiveness of hepatoma cells to leukemia inhibitory factor. Hepatology.

[22] Okamura Y (2010). Leukemia inhibitory factor receptor (LIFR) is detected as a novel suppressor gene of hepatocellular carcinoma using double-combination array. Cancer Lett.

[23] Zhao J (2016). A novel long noncoding RNA-LOWEG is low expressed in gastric cancer and acts as a tumor suppressor by inhibiting cell invasion. J Cancer Res Clin Oncol.

[24] Zhao H, Wang J, S. Tony To (2015). The phosphatidylinositol 3-kinase/Akt and c-Jun N-terminal kinase signaling in cancer: alliance or contradiction? (review). Int J Oncol.

[25] Tavazoie S (2008). Endogenous human microRNAs that suppress breast cancer metastasis. Nature.

[26] Wang J (2017). miR-629-3p may serve as a novel biomarker and potential therapeutic target for lung metastases of triple-negative breast cancer. Breast Cancer Res.

[27] Wang Y (2017). Systematic characterization of A-to-I RNA editing hotspots in microRNAs across human cancers. Genome Res.

[28] Chen D (2012). LIFR is a breast cancer metastasis suppressor upstream of the Hippo-YAP pathway and a prognostic marker. Nat Med.

[29] Wolf J (2016). Different soluble forms of the Interleukin-6 family signal transducer gp130 fine-tune the blockade of Interleukin-6 trans-signaling. J Biol Chem.

[30] Kosfeld A (2017). Mutations in the leukemia inhibitory factor receptor (LIFR) gene and Lifr deficiency cause urinary tract malformations. Hum Mol Genet.

[31] Luo Q (2015). LIFR functions as a metastasis suppressor in hepatocellular carcinoma by negatively regulating phosphoinositide 3-kinase/AKT pathway. Carcinogenesis.

[32] Zhen Y (2017). miR-374a-CCND1-pPI3K/AKT-c-JUN feedback loop modulated by PDCD4 suppresses cell growth, metastasis, and sensitizes nasopharyngeal carcinoma to cisplatin. Oncogene.

[33] Peng Y (2016). Direct regulation of FOXK1 by C-Jun promotes proliferation, invasion and metastasis in gastric cancer cells. Cell Death Dis.

[34] Sui H (2014). miR200c attenuates P-gp-mediated MDR and metastasis by targeting JNK2/c-Jun signaling pathway in colorectal cancer. Mol Cancer Ther.

[35] Zhang Y (2007). Critical role of c-Jun overexpression in liver metastasis of human breast cancer xenograft model. BMC Cancer.

[36] Shao J (2013). COP1 and GSK3β cooperate to promote c-Jun degradation and inhibit breast cancer cell tumorigenesis. Neoplasia.

[37] Gokulnath M (2017). Transforming growth factor-β1 regulation of ATF-3, c-Jun and JunB proteins for activation of matrix metalloproteinase-13 gene in human breast cancer cells. Int J Biol Macromol.

[38] Johnson G, Nakamura K (2007). The c-Jun kinase/stress-activated pathway: regulation, function and role in human disease. Biochim Biophys Acta.

[39] Gustems M (2014). C-Jun/c-Fos heterodimers regulate cellular genes via a newly identified class of methylated DNA sequence motifs. Nucleic Acids Res.

[40] Zhao M (2016). miR-3188 regulates nasopharyngeal carcinoma proliferation and chemosensitivity through a FOXO1-modulated positive feedback loop with mTOR-p-PI3K/AKT-c-JUN. Nat Commun.

