# Supplementary material for: miR-589 promotes gastric cancer aggressiveness by a LIFR-PI3K/AKT-c-Jun regulatory feedback loop
Source: J Exp Clin Cancer Res. 2018 Jul 16;37:152. doi: 10.1186/s13046-018-0821-4 (PMC6048856; doi:10.1186/s13046-018-0821-4)
Supplement: Supplementary file 1 — Supplementary materials and methods. (DOC 82 kb) [file 13046_2018_821_MOESM1_ESM.doc]

**Supplementary Materials and Methods**

**RNA isolation, reverse transcription, and quantitative real-time PCR**

Total RNA was extracted using Trizol reagent (Invitrogen). To quantitate LIFR and c-Jun expression, total RNA was polyadenylated and underwent reverse transcription using PrimeScript™ RT Master Mix (TaKaRa, Dalian, China). Real-time PCR was carried out using a SYBR® Premix Ex TaqTM II (TaKaRa, Dalian, China) on an ABI 7500HT system. To quantitate miR-589 expression, total RNA was polyadenylated and underwent reverse transcription using an NCode miRNA First-Strand cDNA Synthesis kit (Invitrogen). Real-time PCR was carried out using an SYBR Green PCR master mix (Applied Biosystems; Foster City, Calif, USA) on an ABI 7500HT system. *GAPDH* or *U6* snRNA were used as an endogenous control. All samples were normalized to internal controls, and fold changes were calculated through relative quantification (2-ΔΔCT). The primers used are shown in Supplementary Table S2.

**Cell migration, metastasis and invasion analysis**

Cell migration was determined using 24-well transwell chambers with 8 μm pore size polycarbonate membranes (Corning Star; Cambridge, Mass, USA). 1 × 10^5^ cells were seeded on the top side of the membrane. The bottom chamber was prepared using 10% FBS as a chemoattractant. Cells were allowed to migrate through the porous membrane for 20 h at 37°C. The cells that stuck to the lower surface of the membrane were treated with a fixation/staining solution (0.1% crystal violet, 1% formalin, and 20% ethanol) for visualization. The cells were counted under a microscope in 5 randomly selected fields (original magnification, ×200). At least 4 chambers from 3 different experiments were analyzed.

Cell invasion was determined using 24-well transwell chambers with 8 μm pore size polycarbonate membranes (Corning Star; Cambridge, Mass, USA). 2 × 10^5^ cells were seeded on the top side of the membrane pre-coated with Matrigel (CORNING, Jiangsu, China) in RPMI1640 medium without serum. The incubation, fixation and quantification were performed as described.

**Wound healing assay**

Confluent monolayers of cells were maintained in serum-containing growth medium for at least 6 d and then in serum-free medium for 24 h. A 200-μL plastic pipette tip was used to scratch the monolayers. The wounded cells were then cultured in a serum-free medium for an additional 48 h and photographed under an inverted phase contrast microscope. Three different points were marked on the plate, and the distance between each point and the edge of the scratch wound was measured before and after cell migration. The mean migration distance (μm) was calculated by subtracting the length after 48 h from that at 0 h. The result was expressed as a migration index, ie, the distance migrated by treated cells compared with the distance migrated by control cells. Experiments were carried out in triplicate and repeated at least 5 times.

**Tumor metastasis assays**

To determine the lung homing potential of cancer cells in vivo, we injected 5 × 10^6^ LV-miR-589-transfected cells and LV-control-transfected cells into nude mice (n = 8/group) through the tail vein. Whole-body optical images were obtained to monitor primary tumor growth and the formation of metastatic lesions. The mice were all sacrificed 8 weeks later, at which time individual organs were removed and metastatic tissue was analyzed using hematoxylin and eosin (H & E) staining.

**Lentiviruses transfection assay**

Lentiviruses overexpressing human miR-589 and empty vector were built by Suzhou GenePharma, Co. Ltd. (Suzhou, China). For lentiviral transfection of MGC803 cells, 100 multiplicity of infection LV-miR-589 or empty vector lentiviruses were added to a well containing 5x10^4^ cells, medium and 8 µg/ml polybrene. After 24 h of incubation, transfected cells were selected with 2 µg/ml puromycin (Sigma-Aldrich). Empty vector lentivirus was used as a control. Selected cells were maintained in growth medium with 0.5 µg/ml puromycin.

**Luciferase reporter assay**

LIFR was predicted to be directly regulated by miR-589 using TargetScan software. A 940-bp fragment of LIFR 3’UTR amplified by PCR primers was cloned into psiCHECK-2 vectors (named wt). The primers were 5’-ATACAGCCGCTCGAGCCCAGGCAGTGGTGAT-3’ (forward) and 5’ AAATATGCGGCCGCAGGGAACAAGGCAGGA-3’ (reverse). Site-directed mutagenesis of the miR-589 binding site in the LIFR 3'UTR (named mt) was performed using GeneTailor Site-Directed Mutagenesis System (Invitrogen). For reporter assays, wt or mt vector and the control vector psiCHECK-2 vector were cotransfected into BGC823, MGC803 and 293T cells with miR-589 mimics. Luciferase activity was measured at 48 h after transfection using the Dual-Luciferase Reporter Assay System (Promega Corporation, Madison, WI, USA).

To generate a miR-589 promoter vector, a 2,000-bp fragment containing one binding site of c-Jun was PCR-amplified and inserted into a psiCHECK-2 luciferase reporter vector. In addition, c-Jun-binding site mutation vectors were constructed. These psiCHECK-2-derived vector and c-Jun-expressing vectors were cotransfected into 293T or MGC803 and BGC823 cells using Lipofectamine 2,000 Reagent (Invitrogen).

**Chromatin immunoprecipitation assay**

According to the manufacturer’s instructions, ChIP assay was performed to examine whether c-Jun combined to miR-589 promoter by a ChIP assay kit (Millipore, catalog: 17-371). MGC803 and BGC823 cells were firstly fixed with 1% formaldehyde to covalently crosslink proteins to DNA and then chromatin was harvested from the GC cells. Crosslinked DNA was sheared to 200–1,000 base pairs in length with sonication and then subjected to an immmmunoselection process, which required the use of Anti-c-Jun antibody (Abclone). Finally, PCR was used to measure enrichment of DNA fragments in the putative c-Jun-binding sites in the miR-589 promoter on the basis of the specific primers, the primers of site 1 were 5’- GCAGCCTCGACCTTCT-3’ (forward) and 5’- GGTGGGCACCTGTAATC-3’ (reverse); The primers of site 2 were 5’- TGGAGGCAGGGCTTAG -3’ (forward) and 5’- GATTATGGCAGGGAGGAT-3’ (reverse)

**Reference**

**Table S1. Correlation between the clinicopathological features and miR-589 expression**

| **Characteristics** |  | **miR-589 expression** | | |
| --- | --- | --- | --- | --- |
|  | **n(%)** | **miR-589 high** | **miR-589 low** | **P-value** |
| **Age（years）** |  |  |  |  |
| **≥55** | 20(58.82) | 9 | 11 |  |
| **<55** | 14(41.18) | 8 | 6 | 0.486 |
| **Gender** |  |  |  |  |
| **Male** | 21(61.76) | 11 | 10 |  |
| **Female** | 13(38.24) | 6 | 7 | 0.724 |
| **TNM stage** |  |  |  |  |
| **I** | 5(14.71) | 1 | 4 |  |
| **II** | 8(23.53) | 3 | 5 |  |
| **III** | 9(26.47) | 6 | 3 |  |
| **IV** | 12(35.29) | 7 | 5 | 0.078 |
| **Tumor invasion** |  |  |  |  |
| **T1+T2** | 15(44.12) | 4 | 11 |  |
| **T3+T4** | 19(55.88) | 13 | 6 | 0.016 |
| **Lymph node metastasis** |  |  |  |  |
| **N0** | 19(55.88) | 8 | 11 |  |
| **N1** | 15(44.12) | 9 | 6 | 0.300 |
| **Distant metastasis** |  |  |  |  |
| **M0** | 24(70.59) | 8 | 16 |  |
| **M1** | 10(29.41) | 9 | 1 | 0.003 |
| **Tumor differentiation** |  |  |  |  |
| **Well** | 7(15.69) | 4 | 3 |  |
| **Moderate** | 14(45.75) | 6 | 8 |  |
| **Poor** | 13(38.56) | 7 | 6 | 0.777 |

**Table S2.** RT-PCR primer sequences for human genes

| **Gene name Forward primer Reverse primer Product length** |
| --- |
| **LIFR TGGAACGACAGGGGTTCAGT GAGTTGTGTTGTGGGTCACTAA 112** |
| **c-Jun CCAAAGGATAGTGCGATGTTT CTGTCCCTCTCCACTGCAAC 62** |
| **GAPDH AAGGTCGGAGTCAACGGATTTG CCATGGGTGGAATCATATTGGAA 159** |
